# Odontological Society of Pennsylvania

**Published:** 1896-11

**Authors:** 

**Affiliations:** Odontological Society of Pennsylvania


### ODONTOLOGICAL SOCIETY OF PENNSYLVANIA.

THE regular monthly meeting of the Odontological Society of Pennsylvania was held at 1415 Walnut Street, October 10, 1896, the President, Dr. C. N. Peirce, in the chair.

The routine business having been concluded, the subject that engaged the attention of the September meeting, that of the action of the State Society at Bellefonte, Pa., was again introduced by Dr. D. D. Smith. He began his remarks by stating: I was not present at the last meeting of this Society, and have just now learned of the action then taken in relation to the reported irregularities at the State Dental Society at its meeting in July last. The matter came to my notice on a very hot day last summer, while on the streets of Philadelphia, and it seems to have been the subject of conversation universally since that time. I do not know that its odor gets any better as the weather grows cooler.

The resolutions which were adopted by this Society in virtue of its being a part of the State Society, as recorded in your minutes fail, in my judgment, to meet the exigencies of the case. All men hearing of such irregularities, as reported to have been committed, would certainly exhibit abhorrence of them, as they have done. No professional man, I may say no man, except he be a politician, thoroughly steeped in the ways of political chicanery, could possibly approve the reports that have come to us, and I, for one, as a member of the profession, feel that a stain has been cast upon us. I do not know that I feel that my private character has sustained injury, but we are suffering as a society and profession from these reports widely circulated, and especially is this true in Philadelphia, and as this is one of the oldest and most influential societies in the State, why should it not take the initiative.

That we may place ourselves, as a Society, on record as not only abhorring such practices, but also as being ready and anxious to discern and punish them, I now offer the following resolution:

*Resolved*, That it is the sense of the Odontological Society of Pennsylvania, as expressed in this meeting, that a committee of three be appointed to investigate and officially report at the next regular meeting of the Society, upon the alleged irregularities in connection with the ballot taken for the two vacancies in the State Board of Examiners at the annual meeting of the Pennsylvania State Dental Society, held at Bellefonte, July, 1896.

A bare statement of the reports, it seems to me, is sufficient for the adoption of something of this nature, and will be all that is required as the action of this Society, looking to a further investigation by the proper authorities. It is said, and I presume truthfully, for I had it directly from our president, that in the official ballot for one of the gentlemen named by the council for examiner, an office which has been created by the Legislature of Pennsylvania, seven ballots only were counted, while eighteen afterwards affirmed that they cast their ballots for him. It becomes us as members of the Odontological Society of Pennsylvania to put the seal of our condemnation on all such unprofessional methods, and place the matter, if need be, into the hands of the legislature for investigation.

Dr. Fellows seconded the resolution.

The president stated the substance of the resolution, and invited discussion.

*Dr. Truman.*—I should like to inquire of the mover of this resolution how the information desired is to be obtained. The facts all seem to lie within the council of the State Society. Our president is a member of that body, and he should be able to give us information in regard to this. I am not in favor of appointing a committee unless that committee can act. I met one of the tellers the other day, and one whom we all respect, and it was his earnest desire that the council should investigate the matter at once. I hold this body largely responsible, for when the opportunity was in its hands for correction, at the State meeting, nothing seems to have been done. My reports are all second hand, and I may do them injustice, hence the necessity for an early and thorough investigation.

*Dr. Head.*—Dr. Truman believes the council condones the offence, then why does he affirm that the council should be permitted to investigate it?

*Dr. Truman.*—I am not opposed to this committee. I only desire information as to the course proposed to pursue to acquire the facts desired.

*Dr. Boice.*—The purpose of making a report is that we may have some intelligence. We are working in the dark, and every one seems afraid to talk. I was the man who raised the point that only seven instead of eighteen of the registered votes had been returned. In denouncing it, I called, "Mr. President, a privileged question! A privileged question!" But the same methods that had prevailed all through the meeting were carried to the close, and "Adjourned! adjourned!" was called, and this is why it is necessary for us to undertake the investigation.

*President Peirce.*—An investigation can give us an official report of just what transpired. That much it can do. We have the resolutions condemning the action without any statement of what that action was.

*Dr. D. D. Smith.*—An earnest committee could accomplish much in a month and secure information upon this matter, as it is known who the gentlemen are that voted for the candidates in question. Many things now dark can be made clear, and a report can be made which will form a basis for future action if it be decided that such action is advisable.

*Dr. Broomell.*—I cannot understand why we, as a subordinate body, should take action for the State Society. If the charges must stand for a year I cannot see how we can act. I know the tellers are very anxious to have an investigation made, and, of course, I favor the matter if it can be carried through.

*Dr. Truman.*—I am of the opinion that this Society has the undoubted right to investigate. It is a matter that certainly deeply interests the dental profession in this State, and must, to a large extent, the entire States of the Union. It is not and cannot be made a mere local affair, for it involves questions that may arise in any State. The question I asked was not for the purpose of antagonizing the resolution, but to gain information as to the proposed course of procedure. If a correct history of the matter be the aim, it is well, and the report, when made, should go upon the minutes and be published in the proceedings.

The resolution was then unanimously adopted.

The president appointed Drs. D. D. Smith, Truman, and Culver as the committee.

Adjourned.

Joseph Head, M.D., D.D.S.,
*Editor Odontological Society of Pennsylvania.*

